# Simultaneous surgical treatment for esophagogastric junctional cancer and splenic artery aneurysm resection with spleen preservation using fluorescence imaging: a case report

**DOI:** 10.1186/s40792-019-0602-0

**Published:** 2019-03-25

**Authors:** Mamoru Miyasaka, Yuma Ebihara, Yoshiyuki Yamamura, Kimitaka Tanaka, Yoshitsugu Nakanishi, Toshimichi Asano, Takehiro Noji, Yo Kurashima, Toru Nakamura, Soichi Murakami, Takahiro Tsuchikawa, Keisuke Okamura, Toshiaki Shichinohe, Satoshi Hirano

**Affiliations:** Department of Gastroenterological Surgery II, Hokkaido University Faculty of Medicine, Kita 15 Nishi 7, Kita-ku, Sapporo, Hokkaido 060-8638 Japan

**Keywords:** Esophagogastric junctional cancer, Fluorescence imaging, Splenic artery aneurysm

## Abstract

**Background:**

Recently, minimally invasive esophagectomy and gastrectomy for esophagogastric junctional (EGJ) cancer using either thoracoscopy or laparoscopy are frequently performed. In the past decade, minimally invasive surgery with laparoscopy for splenic artery aneurysm (SAA) has also been reported. However, patients with both EGJ cancer and SAA are rare.

**Case presentation:**

A 66-year-old man, who complained of upper abdominal pain, was found to have esophagogastric junctional (EGJ) tumor. He was diagnosed as having Siewert type II adenocarcinoma. In a computed tomography (CT) scan before surgery, a 10-mm aneurysm in the splenic artery was found. Thus, we performed laparo- and thoracoscopic proximal gastrectomy and lower esophagectomy for EGJ cancer and splenic artery aneurysm (SAA) resection with spleen preservation using fluorescence imaging.

We confirmed sufficient blood supply to the spleen after surgery with a postoperative CT scan. The blood supply to the spleen was suspected to be from the great pancreatic artery via the pancreas and from the omental branches of the left gastroepiploic artery via the omental artery.

**Conclusion:**

Simultaneous surgery for EGJ cancer and SAA is rare due to its potential risk, but evaluation of the blood supply for the spleen by using fluorescence imaging can be useful for this procedure.

## Background

Recently, the incidence of esophagogastric junctional (EGJ) cancer has increased [1]. Minimally invasive esophagectomy and gastrectomy for EGJ cancer using either thoracoscopy or laparoscopy are frequently performed to reduce postoperative complications [2]. In the past decade, minimally invasive surgery with laparoscopy for splenic artery aneurysm (SAA) has also been reported [3]. Herein, we describe the case of a patient with EGJ cancer and SAA who underwent simultaneous laparoscopic proximal gastrectomy and SAA resection with spleen preservation using fluorescence imaging.

## Case presentation

**Fig. 1:**
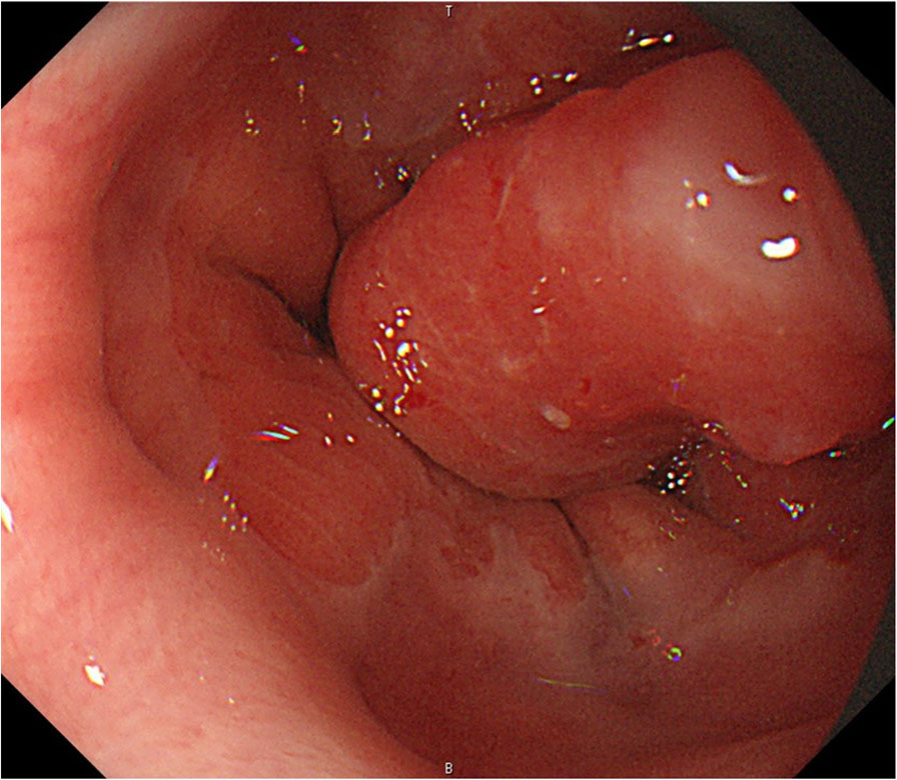
Upper gastrointestinal endoscopy. Endoscopy reveals a 15-mm type I tumor at the esophagogastric junction

A 66-year-old man, who complained of upper abdominal pain, was found to have EGJ tumor by endoscopy (Fig. 1). He was diagnosed as having Siewert type II adenocarcinoma (E=G, cT1b, cN0, cM0, cStage I) [4]. We planned to perform lower esophagectomy and proximal gastrectomy with double tract reconstruction.

**Fig. 2:**
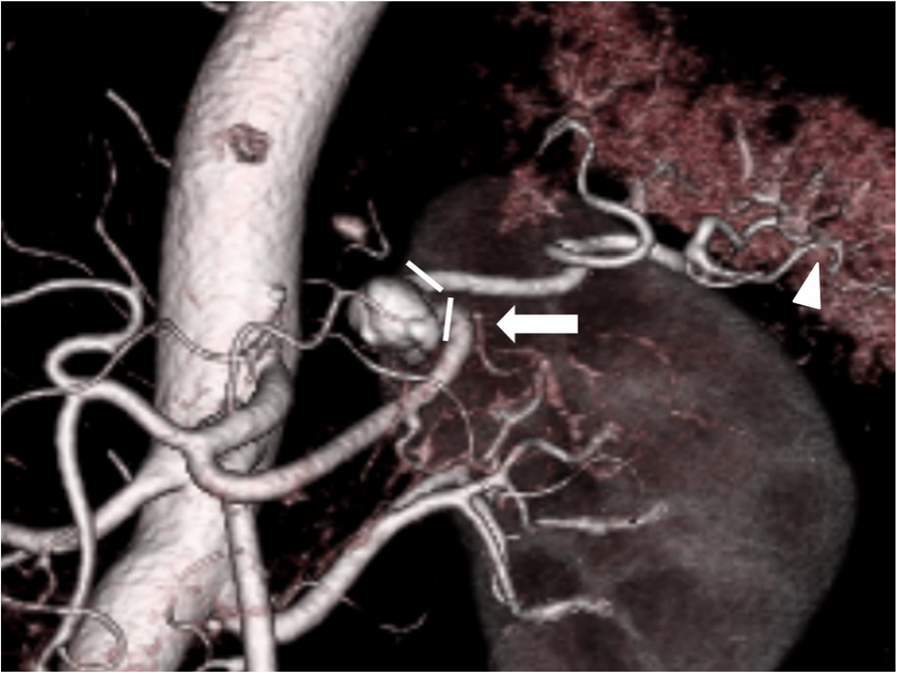
Preoperative enhanced computed tomography. Enhanced computed tomography reveals a 10-mm aneurysm in the middle third of the splenic artery. *White triangle: omental branches of the left gastroepiploic artery via the omental artery; white arrow: the great pancreatic artery via the pancreas; white lines: the range where the splenic artery was resected

In the computed tomography (CT) scan before surgery, a 10-mm aneurysm in the middle third of the splenic artery was found (Fig. 2). Due to the anatomical location of the aneurysm, endovascular treatment was not considered due to recanalization and coil migration. The size of the aneurysm was not a high risk of rupture [5], but we concerned about the possibility of SAA rupture due to postoperative pancreatic fistula (PF) associated with suprapancreatic lymph node dissection such as station 11p and 11d. We thought that spleen blood flow could be preserved even after SAA resection by preoperative CT scan and unnecessary invasive procedures could be avoided by simultaneous surgery. However, during simultaneous proximal gastrectomy and SAA resections, it was not clear whether there was sufficient blood supply to the spleen. Thus, we performed the surgery with a minimally invasive abdominal and left thoracic approach (MALTA) while continuously examining for the presence of blood supply to the spleen using fluorescence imaging.

### Operative procedure

**Fig. 3:**
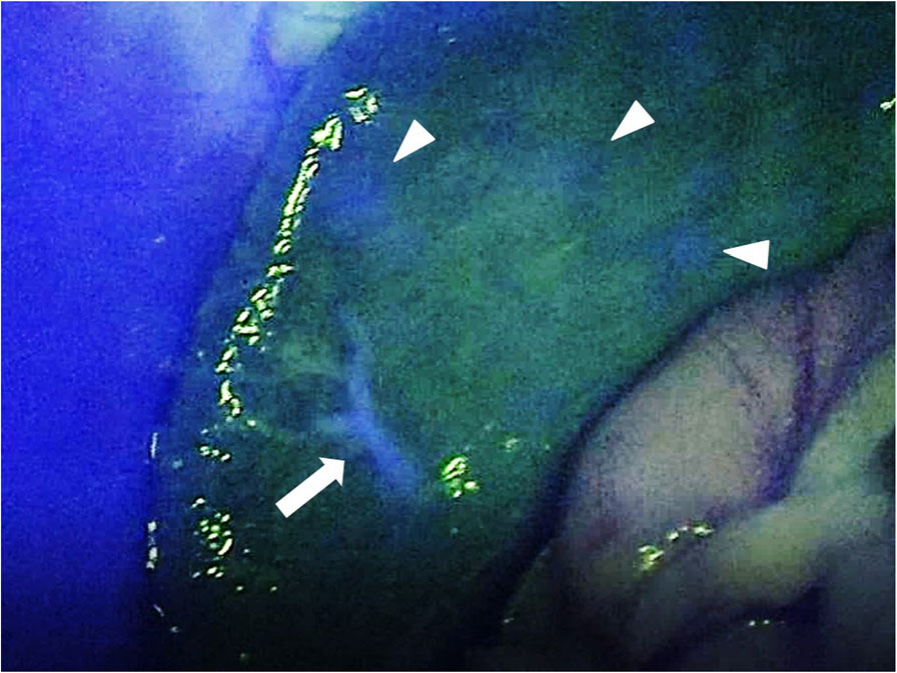
Intraoperative fluorescence imaging. Intraoperative fluorescence imaging shows the fluorescing of the spleen after splenic artery aneurysm (SAA) resection. *White triangle: the fluorescent part in the spleen; white arrow: the intrasplenic artery

The patient was placed under general anesthesia and was positioned in reverse Trendelenburg with the left side of the upper body lifted and the legs spread. Laparo- and thoracoscopic proximal gastrectomy and lower esophagectomy with D1+ lymph node dissection and double tract reconstruction were performed. First, we resected the SAA. Next, laparoscopic proximal gastrectomy was performed using five ports. Four ports were inserted into the thoracic cavity through the 9th, 10th (× 2), and 11th intercostal spaces, with the patient in the same body position. Lower thoracoscopic esophagectomy and lymph node dissection of the lower mediastinum was performed under artificial pneumothorax. The lower esophagus was resected under the thoracoscopic view to ensure an adequate margin. SAA located at the distal side of the branch of the great pancreatic artery (Fig. 2), so we clamped the splenic artery to preserve the great pancreatic artery temporally. Then, we injected 5 mg of indocyanine green (ICG) intravenously to the patient. Blood supply to the spleen was confirmed using fluorescence imaging (Fig. 3). Therefore, we resected the SAA without splenectomy (preserving the great pancreatic artery and the spleen). Intrathoracic esophagojejunostomy was performed with a transoral anvil (OrVil™) and a circular stapler (EEA25™) using the laparo- and thoracoscopic techniques. In addition, an oblique jejunogastrostomy for double tract reconstruction was performed. The operative time was 362 min, and there was no blood loss.

### Postoperative course

**Fig. 4:**
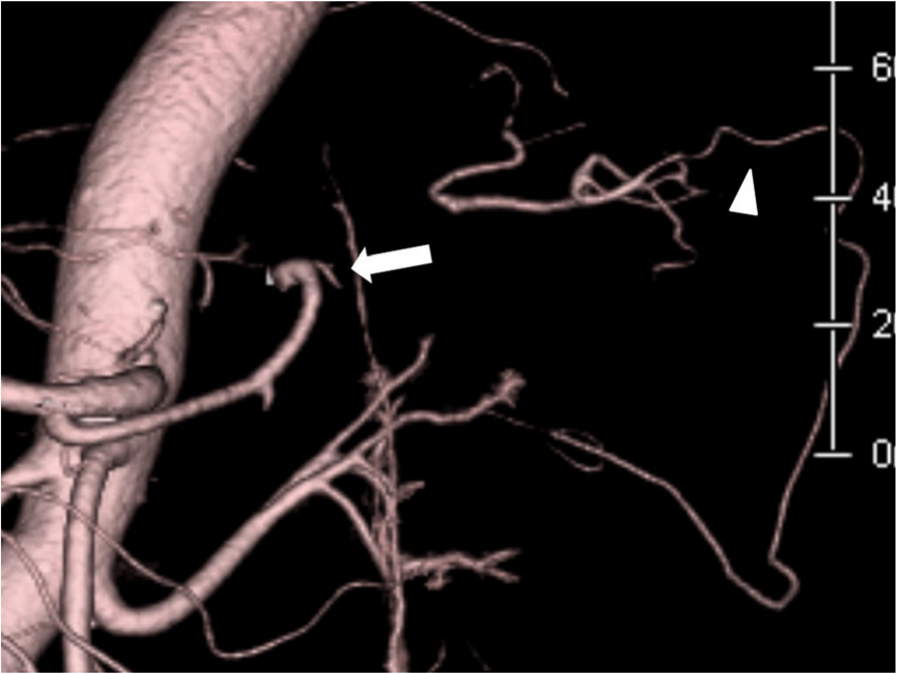
Postoperative enhanced computed tomography. Postoperative enhanced computed tomography reveals the arteries that could supply blood to the spleen.*White triangle: omental branches of the left gastroepiploic artery via the omental artery; white arrow: the great pancreatic artery via the pancreas

Histopathological diagnosis of the tumor was adenocarcinoma with enteroblastic differentiation (pTIb, pN1, pStage IIB according to the UICC Classification, 8th edition,) and with clear margins. Oral intake was resumed on postoperative day 5. Postoperative CT scan revealed the preservation of the great pancreatic artery and omental branches of the left gastroepiploic artery (Fig. 4). There was no splenic abscess due to ischemia or other complications. The patient’s length of hospital stay was about a month.

## Discussion

In recent years, the incidence of EGJ adenocarcinoma has increased [1]. Gastroesophageal reflux disease, smoking, and obesity are regarded as risk factors for EGJ adenocarcinoma [6]. Surgery is the center of curatively intended treatment of EGJ cancer. However, the optimal surgical strategy for this disease remains controversial [7]. For Siewert type II adenocarcinomas in EGJ cancer, lower esophagectomy and proximal gastrectomy are often performed.

Regarding SAA, the incidence of visceral aneurysms has been reported to be between 0.01 and 2% in the general population [5]. However, in recent years, patients are diagnosed more frequently, despite being nonsymptomatic, due to the advancement of medical imaging equipment. For SAA with diameters exceeding 2 cm, the indication of treatment depends on various factors [5].

Laparotomy was a common treatment of SAA in the past, but minimally invasive treatment has been increasing nowadays [3, 8]. Moreover, treatment options for SAA include elective splenectomy, aneurysm ligation or resection, and endovascular transcatheter embolization or stent grafting. There are few invasions with endovascular treatment, but SAA in the proximal splenic artery or meandering arteries may not be suitable for endovascular treatment [8]. In the present case, the size of the aneurysm was not a high risk of rupture. If the blood supply to the spleen was inadequate after SAA resection, there was a risk to add unnecessary invasive procedure of splenectomy to the proximal gastrectomy for EGJ cancer. However, because the aneurysm was located slightly proximal to the splenic artery, there was a possibility that endovascular treatment was not enough. There was also a risk of SAA rupture due to postoperative PF. Considering these risks, we could simultaneously perform laparoscopic SAA resection and avoid unnecessary invasive procedures.

A potential problem in this simultaneous surgery is the blood supply to the spleen. In proximal gastrectomy, the short and posterior gastric arteries are divided. They are thought to be collateral circulation pathways when blood supply in the main trunk of the splenic artery is blocked [9]. However, in the present case, we could assess the blood supply to the spleen using fluorescence imaging after proximal gastrectomy (Fig. 3). Unnecessary splenectomy can be avoided when using such a method. In this case, blood supply to the spleen was suspected to be from the great pancreatic artery via the pancreas and from the omental branches of the left gastroepiploic artery via the omental artery (Fig. 4). As a result, there was no appearance of splenic infarction or delayed abscess after the surgery.

To the best of our knowledge, this is the first report of simultaneous surgery for EGJ cancer and SAA in the English literature. Simultaneous surgery has the advantage of reducing invasive procedures to the patient. In laparoscopic proximal gastrectomy with SAA resection, we can avoid concomitant splenectomy if the great pancreatic artery and omental branches from the left gastroepiploic artery can be preserved. Evaluation of blood supply for the spleen using fluorescence imaging is useful for intraoperative decision-making.

## Conclusions

To the best of our knowledge, this report describes the first case of simultaneous surgery for EGJ cancer and SAA with spleen preservation using fluorescence imaging. The management of EGJ cancer and SAA continue to be controversial. We presented a feasible option of a successful simultaneous laparoscopic and thoracoscopic surgery. It may be considered as one of the treatment options for patients with EGJ cancer and SAA.

## Abbreviations

CT: Computed tomography
EGJ: Esophagogastric junctional
ICG: Indocyanine green
MALTA: Minimally invasive abdominal and left thoracic approach
PF: Pancreatic fistula
SAA: Splenic artery aneurysm
